# Endocrine Responses to Various 1 × 1 Small-Sided Games in Youth Soccer Players

**DOI:** 10.3390/ijerph16244974

**Published:** 2019-12-06

**Authors:** Paweł Chmura, Tomasz Podgórski, Marek Konefał, Andrzej Rokita, Jan Chmura, Marcin Andrzejewski

**Affiliations:** 1 Department of Team Games, University School of Physical Education, 51-612 Wrocław, Poland; pawel.chmura@awf.wroc.pl (P.C.); andrzej.rokita@awf.wroc.pl (A.R.); 2 Department of Physiology and Biochemistry, Poznań University of Physical Education, 61-871 Poznań, Poland; podgorski@awf.poznan.pl; 3 Department of Biological and Motor Sport Bases, University School of Physical Education, 51-612 Wrocław, Poland; jan.chmura@awf.wroc.pl; 4 Department of Methodology of Recreation, Poznań University of Physical Education, 61-871 Poznań, Poland; andrzejewski@awf.poznan.pl

**Keywords:** small-sided games, cortisol, testosterone, T/C ratio, hormones, soccer

## Abstract

The aim of this study was to determine relationships between repeated 1 × 1 small-sided games (SSGs) (variable duration, constant work-to-rest ratio) and the concentration of steroid hormones and characteristic fatigue markers in youth soccer players. Eighteen young male soccer players were assigned at random to two experimental groups: E1—undertaking a six 30 s one-on-one SSGs with a 2 min rest period; and E2—playing six 45 s SSGs with a 3 min rest interval. Capillary blood was collected from the players at rest, after the last game, and 15 and 30 min after the exercise protocol. The variables assessed included serum cortisol (C), free testosterone (FT) and total testosterone (TT). An effect was observed between the measurement times (TT (F = 15.26, *p* ≤ 0.0001), FT (F = 6.86, *p* = 0.0006)). In terms of cortisol (C) levels, no interactions or effect between the studied groups were revealed, but an interaction was found (F = 4.01, *p* = 0.0126) and the effect appeared between the measurement times (F = 11.16, *p* ≤ 0.0001). The study results show that in all likelihood, longer rest intervals in repeated 30 s 1 × 1 SSGs can reduce catabolic reactions and hence the risk of overtraining in youth soccer players.

## 1. Introduction

Testosterone and cortisol play a major role in the biochemical assessment of athletes. Testosterone (T) is the most significant blood androgenic hormone. In males, testosterone is synthesized primarily in Leydig cells in the testicles and is responsible for the appearance of male secondary sex characteristics, having typical anabolic effects on the human body—in particular, on protein synthesis [1]. At rest, approximately 99% of all testosterone is bound with sex hormone-binding globulin and albumins [2]. At a given moment, only 1% of testosterone is free, and free testosterone (FT) can have an anabolic effect, increasing strength, muscle mass, or bone density [3]. Cortisol (C), synthesized in the zona fasciculata of the adrenal cortex, evokes opposite responses. It has anti-inflammatory properties, stimulates gluconeogenesis and glycogenolysis in the liver, and increases the concentration of blood glucose. Due to its inhibition of amino acid uptake by muscles and decreased protein synthesis, cortisol is considered a catabolic hormone [4].

Sport-specific training has a significant impact on blood testosterone [5]. The effect of regular exercise on testosterone is less clear but, in this case, the intensity and duration of exercise also play a significant role. Short-duration exercises of high intensity, along with resistance training, affect testosterone levels [6,7]. On the other hand, studies of endurance sport athletes have revealed lower testosterone levels after exercise [8,9,10].

Researchers have indicated possible correlations between exercise and cortisol secretion [11]. The main determinants include the intensity, duration and type of exercise, and the environment (e.g., elevation and temperature) [9,11,12]. Brisswalter, Collardeau and Rene [4] showed that plasma cortisol increases after acute exercise exceeding 60% of VO_2_max, and also after intensive resistance exercise [4]. Studies also revealed that engaging in short-duration, high-intensity exercise leads to a significant rise in the cortisol level in sedentary controls and trained athletes [13,14]. Such a measurement of cortisol level can thus be used as an indicator of physical stress.

It appears that cortisol can also serve as a marker of exercise intensity. However, given its sensitivity to certain stimuli, it might be more advantageous to refer to the testosterone/cortisol ratio (T/C) as an indicator of anabolic/catabolic balance, psychophysical stress and fatigue in athletes, and of directions of adjustment of current training loads [7,15,16]. T/C is also perceived as an early indicator of overtraining, capable of eliminating an athlete from competition for weeks or even months [17]. At present, two commonly used T/C ratios are total testosterone/cortisol (TT/C) and free testosterone/cortisol (FT/C). Low TT/C and FT/C ratios are suggested as markers of the fatigue and overtraining syndrome, but only a very low value of this ratio is useful [18].

Small-sided games (e.g., 1 × 1) are popular with coaches, as they are deemed useful drills for concurrent training of physical, technical, and tactical skills. Several authors have claimed that, if designed properly, small-sided games (SSGs) can replicate the physiological and motor demands as well as technical and tactical demands of real match play [19]. However, their greatest benefits are related to physical fitness. For instance, SSGs have been confirmed as having an effectiveness equal to that of high-intensity running intervals when it comes to generating elevated heart rates (90% HRmax) and blood lactate concentrations, improving sport-specific endurance [20,21]. Owen, Twist and Ford [22] confirmed that intensity as measured by heart rate (HR) in the course of a 1 × 1 game corresponds with exercise intensity attained during a championship match. The effectiveness of small-sided games used as training interventions has also been demonstrated in relation to significant improvements in players’ aerobic fitness [23]. Several recent studies have also investigated the acute effects of SSGs on players’ anaerobic fitness, speed endurance [24], and agility [25]. Furthermore, Sparkes et al. [26] rated the recovery patterns of neuromuscular function and perturbations in physiological responses and mood for up to 24 h. Some authors highlighted the agility demands of SSGs, and the effects of changing the number of players or the augmentation of games on the physiological and mental demands of these exercises [25,27,28]. Additionally, SSGs are successfully used to overload the anaerobic system through repeated accelerations [24].

The programming of training during a season can prove challenging for coaches, given the requirement that they prescribe loads maximizing players’ positive physiological and biochemical adaptation, while avoiding overtraining and injury. There is a particular lack of research on players’ fatigue and recovery in an SSG context, and on the implications that this has for recovery strategies. Equally, hormonal responses could provide insights into the internal loading and adaptation to stressors associated with SSGs and matches. The levels of various hormones could also provide information on training and recovery protocols for a team as a whole, and for players in their specific positions. However, studies so far have failed to provide data on the hormonal responses of players of 1 × 1 small-sided games of various duration, notwithstanding the fact that such data would allow for more effective monitoring and individualization of training loads. Therefore, the aim of this study was to determine the relationship between repeated 1 × 1 SSGs (variable duration, constant work-to-rest ratio) and levels of steroid hormones and characteristic fatigue markers in youth soccer players.

## 2. Materials and Methods

### 2.1. The Experimental Approach to the Problem

The main assumption of the study was that repeated 1 × 1 SSGs of variable duration but constant work-to-rest ratios will affect the selected hormonal markers: testosterone (TT), free testosterone (FT) and cortisol (C) as well as the ratios between them, i.e., (TT/C) and (FT/C). In this way, we studied the stress levels provoked by SSGs in young competitive soccer players. Two groups of players participated in six different repeated 1 × 1 SSGs. Their blood was sampled at rest, prior to the start of the SSGs, after the last game, and 15 and 30 min after the whole exercise protocol. After a game, players recovered passively (by sitting). Statistically significant differences in the examined biochemical markers between exercise duration and responses were expected.

### 2.2. Participants

Eighteen youth male soccer players belonging to an academy team from a First-Division professional club in Poland participated in the study. The participants’ age and training experience are presented in Table 1, along with anthropometric characteristics. The participants had significant soccer training experience (of 8.7 ± 3.35 years) and competed at the highest level in their age category (under 18). On average, the players trained 5 times a week and played one official match. Parental or guardian consent was obtained from participants under 18 years of age. The participants were fully informed about the study procedures and the experimental risk.

The study was approved by the Research Ethics Committee (no. 20/2017) of the University School of Physical Education in Wroclaw and was consistent with institutional ethical requirements for human experimentation under the Declaration of Helsinki.

### 2.3. Procedures

The study’s combination of 30 and 45 s 1 × 1 games in 6 repetitions has been frequently applied in sport-specific training [29]. Match analyses show that players engage in one-on-one plays every 3–4 min during a soccer game [30]. The playing time/rest time ratio applied in the present study was 1:4 [22]. In line with other research, the players were divided at random into two experimental groups: E1 (*n* = 10)—undertaking an SSG program of shorter-duration work and rest, i.e., playing six 30 s one-on-one (1 × 1) SSGs with a 2 min rest interval between each game; and E2 (*n* = 8)—undertaking an SSG protocol in which six 45 s (1 × 1) SSGs were played with 3 min rest periods between the games. The soccer players were asked to put in the maximum effort during the games. Moreover, verbal encouragement was given by the coaches throughout the games.

The matches were played on a 10 × 15 m synthetic grass pitch surrounded by boards. The tests were conducted in the morning (10:00 to 12:00) in stable weather conditions (sunny weather with almost no wind and an ambient temperature of 10–12 °C). The pitch was dry during all training sessions.

### 2.4. Blood Sampling

Capillary blood was collected from the players at rest, after the last game, and 15 and 30 min after the whole exercise protocol. The samples were drawn from the fingertip of the non-dominant hand using a disposable Medlance^®^ Red lancet-spike (HTL-Zone, Berlin, Germany) with a 1.5 mm blade and 2.0 mm penetration depth. In total, 300 µL of capillary blood was collected into a Microvette^®^ CB 300 Z tube (Sarstedt, Oslo, Norway) with a clotting activator, and the separated serum steroid hormone concentration was analyzed. Concentrations of serum cortisol (C), free testosterone (FT) and total (TT) testosterone were determined using commercially available ELISA kits (DRG MedTek, Warsaw, Poland). Spectrophotometric measurements of ELISA tests were made on a multi-mode microplate reader (Synergy 2 SIAFRT, BioTek, Winooski, VT, USA).

### 2.5. Statistical Analysis

All data were presented as means and standard deviations (mean ± SD). The normality of variable distribution was checked with the Shapiro–Wilk test. The homogeneity of variance was checked by Bartlett’s test. The data were subject to a multi-way repeated measures analysis of variance (ANOVA) 2 × 4 (groups x measures) to examine changes in the mean hormone values for over the 1 × 1 small-sided games. When a significant effect size was found, a post-hoc Duncan test was performed. To describe differences relating to angular velocities, effect sizes were calculated as differences between means divided by the pooled standard deviation. Using Cohen’s criteria [31], an effect size of ≥0.20 and <0.50 was considered small, ≥0.50 and <0.80 medium, and ≥0.80 large. The level of statistical significance was set at *p* < 0.05. All statistical analyses were made with the use of the STATISTICA ver. 13.1 software package (StatSoft. Inc., Tulsa, OK, USA).

## 3. Results

The players’ anthropometric characteristics and training experience are shown in Table 1. There were no significant differences between the two groups of players with regard to the examined variables.

The statistical analysis of total and free testosterone levels revealed no interactions or effect between the studied groups. However, an effect was observed between the measurement times (TT (F (3, 48) = 15.26, *p* ≤ 0.0001, FT (F (3, 48) = 6.86, *p* = 0.0006) (Table 2). In terms of cortisol (C) levels, no interactions or effect were revealed between the studied groups; however, an interaction was found (F (3, 48) = 4.01, *p* = 0.0126) and the effect appeared between the measurement times (F (3, 48) = 11.16, *p* ≤ 0.0001) (Table 2). The analysis of anabolic/catabolic indicators (TT/C, FT/C) revealed no interactions or effect between the studied groups. However, an effect was again noted between the measurement times (TT/C (F (3, 48) = 7.45, (*p* = 0.0003), FT/C (F (3, 48) = 5.11, *p* = 0.0038) (Table 2).

A large effect size for total testosterone appeared between the measurement times: immediately post-exercise and 30 min post-exercise (d = 1.16), as well as at rest and 30 min post-exercise (d = 0.96). Additionally, a medium effect size was found between immediately post-exercise and 15 min post-exercise (d = 0.71) measurements; and in the case of free testosterone between immediately post-exercise and 30 min post-exercise (d = 0.66). In the case of cortisol, a large effect size appeared between the measurements at rest and immediately post-exercise (d = 1.07), and a medium effect size between 15 and 30 min post-exercise (d = 0.54). A large effect size for TT/C was noted between the measurements at rest and 15 min post-exercise (d = 0.93), and at rest and 30 min post-exercise (d = 0.84). A medium effect size for FT/C was found between the measurements at rest and 15 min post-exercise (d = 0.74), as well as at rest and 30 min post-exercise (d = 0.62). In group E1, a large effect size for cortisol concentration was noted between the measurements at 15 min post-exercise and immediately post-exercise (d = 0.85) and at rest (d = 1.70), and 30 min post-exercise and at rest (d = 1.00).

## 4. Discussions

The study attempted to determine the relationship between repeated 1 × 1 SSGs (variable duration, constant work-to-rest ratio) and the concentration of steroid hormones and characteristic fatigue markers in youth soccer players.

In our experiment, we showed that in both groups, i.e., E1 and E2, immediately after exercise, the total testosterone (TT) and free testosterone (FT) levels were the highest after six 1 × 1 SSGs. This can be linked to the higher catecholamine blood level in the players, or to exercise-induced (SSGs) arousal of the sympathetic nervous system [7]. The secretion of testosterone is an indicator of total training stress, rather than of the influence of individual external cues, such as specific running patterns, speed of movement, or distance covered at different intensities. The immediate post-exercise TT levels were significantly higher that the TT levels at rest (both 15 and 30 min post-exercise). It seems that the cause of the observed changes in the immediate post-exercise testosterone secretion may be related to the high intensity of exercise, mental stress, and the levels of catabolic hormones such as cortisol [10].

Additionally, the 1 × 1 SSGs induced changes with regard to total testosterone (TT) in comparison with resting levels. The TT levels at rest were significantly higher than 30 min post-exercise. Keizer, Janssen, Menheere, et al. [32] noted that while long-distance running training was associated with a steady increase in testosterone concentration, and the running of an actual marathon was associated with its decrease commensurate with the distance covered. It thus appears that exercise of excessively high duration may cause a decline in testosterone concentration. Changes in the testosterone concentration were also correlated positively with the peak lactate level after maximum-intensity exercise, whereas cortisol levels were negatively correlated with VO_2_max, which impaired sports performance [33].

Cortisol is a catabolic muscle breakdown steroid hormone known as “the stress hormone”, which has been shown to increase in concentration following exercise in almost all sports, including soccer, regardless of athlete’s age or gender [34,35]. Cortisol is released in response to stress on both a physical and a psychological scale, which accounts for increases in measurement levels of cortisol as determiners of stress experienced in training. Banfi and Dolci [34]; Edwards, Wetzel and Wyner [35]; VanBruggen, Hackney, McMurray, et al. [17]; and Labsy, Prieur, Le Panse, et al. [36] asserted that the nature of soccer and its physical demands should stimulate a change in cortisol levels during training and competition. Some authors indicate that consecutive high-intensity exercises are related to a rising cortisol level [13,14]. Vuorimaa, Ahotupa, Hakkinen, et al. [33] found that continuous exercise of lower intensity (80% VO_2_max) leads to a more limited increase in cortisol concentration than intermittent exercise of high intensity (100% VO_2_max). This may indicate that it is not exercise intensity alone, but also training volume, that may exert the greatest influence on the cortisol level [37]. Abe, Kearns and Sato [38] showed that exercise of low intensity causes only a slight rise in blood cortisol. On the other hand, short sub-maximal anaerobic exercises affect cortisol levels significantly later than testosterone levels [39].

Similar changes were observed in the present study, in which the immediate post-exercise levels of cortisol in both groups (E1, E2) were significantly higher than the resting levels. This was also observed by de Wall [40], who found an increase in cortisol after 4 × 4 and 7 × 7 games as compared with baseline values. de Wall [40] also revealed a lower cortisol level after an 11 × 11 game than at rest.

The duration of rest intervals between exercises may also have a significant impact on hormonal responses in players [41,42]. Rahimi, Rohani and Ebrahimi [42] showed that 120 s rest periods between resistance training sessions were linked to higher testosterone levels and lower cortisol levels than 60 and 90 s rest intervals. This can be evidence of a more beneficial anabolic effect of a longer rest period in this type of exercise, as confirmed in the present study. In group E1, where the rest period between SSGs was shorter, the changes were greater than in group E2, where catabolic processes were dominant. The highest cortisol level was noted 15 min post-exercise in group E1, and this was significantly greater than the levels at rest or immediately post-exercise.

It should also be noted that 30 min post-exercise, the cortisol levels in E1 were significantly higher than at rest. In E2 players, the cortisol levels were similar at different measurement times. Port [43] observed that low exercise intensity leads to non-significant changes in cortisol levels, while an increase in exercise intensity causes a significant rise in cortisol concentration. Additionally, the latter is correlated with exercise-induced lactate accumulation. de Vries, Bernards, de Rooij, et al. [44] explained the delayed return of cortisol concentration to baseline levels in terms of blood glucose decrease and as a possible tissue-mobilized defense against exercise-induced damage.

The TT/C ratio has been reported as an indicator of anabolic/catabolic homeostasis and overtraining [6,34,35,36,37,38,39,40,41,42,43,44,45]. These authors assert that the ratio between these two hormones is known as the free testosterone/cortisol ratio (FT/C), and that free testosterone is defined as testosterone not linked to any proteins. Banfi and Dolci [34], in their study of elite soccer players over a period of two years, provided an interesting insight into the chronic or longitudinal aspects of the TT/C ratio and its relationship with overtraining. Their view is supported by Alexiou and Coutts [46], who suggest that long-term monitoring of training loads may assist coaches in controlling the training process and improving players’ performance. When analyzing the acute response of the TT/C and FT/C ratios to exercise, it is important to note the lack of studies aimed at the monitoring of TT/C and FT/C ratio responses to 1 × 1 soccer SSGs.

The present study shows that TT/C and FT/C concentrations at rest are significantly higher than those measured 15 and 30 min post-exercise. Intensive training of long duration leads to a reduced TT/C ratio. Likewise, Urhausen, Kullmer and Kindermann [47] noted a gradual decline in the TT/C ratio in the course of a 7 week rowing training program, which they saw as indicative of intensified catabolic processes. They also noted that the TT/C ratio increased during recovery, redressing the anabolic/catabolic balance. Similarly, the present study revealed that TT/C and FT/C levels were slightly higher 30 min post-exercise than 15 min post-exercise. A faster immediate post-exercise increase in TT/C and FT/C was found in E2 players taking part in six 45 s 1 × 1 games with 3 min rest intervals. This may suggest that 30 s repeated exercises with a 2 min rest interval enhance catabolism in youth male soccer players. This correlation is most likely due to exercise duration and the shorter rest interval between repeated 1 × 1 games.

Further research is required utilizing repeated measure designs to fully examine endocrine responses and to better identify any changes that occur within a player’s body. To this end, it would be necessary to carry out tests of the same working time in a variable work-to-rest ratio. Owing to the hormone analysis costs, future research into the correlation between cortisol and testosterone and common, more cost-effective measures of internal training loads such as HR, session rating of perceived exertion (sRPE), and blood lactate, or combined measures, should be carried out. Prospective studies should aim to determine the ideal change in TT/C ratio between testing points in order to stimulate maximal physiological adaptation in players without inducing overreaching or chronic overtraining symptoms.

## 5. Practical Applications

The present study is one of the first to report changes in blood testosterone and cortisol concentrations of youth soccer players taking part in 1 × 1 small-sided games. The concentrations of these hormones and obtained ratios are known markers of an athlete’s functional status and should be measured during small-sided games to help coaches individualize the training process as needed. Soccer coaches and sports scientists have to observe changes in these hormones regularly in order to improve players’ performance. To make the measurement of these hormones useful, they should not be isolated but rather timed regularly so that the changes involved may be observed by a medical team. Proper cooperation between the latter and coaches will help maximize players’ performance, while limiting any risk of overtraining.

However, coaches can consider using combined measures for monitoring training and match loads as opposed to endocrine responses only. Questionnaires, external loading data (GPS, time-motion analysis, accelerometers, etc.) and other means of monitoring internal training loads (HR, sRPE, blood lactate, etc.) should all be used to provide a broader picture of the total training load.

## 6. Conclusions

The study results show that, in all likelihood, longer rest intervals in repeated 30 s 1 × 1 SSGs can reduce catabolic reactions and hence the risk of overtraining in youth soccer players. Future research should attempt to reduce exercise duration in repeated 1 × 1 games, while also increasing the work-to-rest ratio.

## Figures and Tables

**Table 1:** Anthropometric characteristics and training experience of youth soccer players (mean ± SD).

Variable	Group E1 (*n* = 10)	Group E2 (*n* = 8)
Age (years)	17.2 ± 0.88	17.3 ± 0.72
Body height (cm)	177.9 ± 5.08	178.9 ± 5.06
Body mass (kg)	70.5 ± 5.55	71.8 ± 5.42
Training experience (years)	8.6 ± 3.35	8.8 ± 3.06

**Table 2:** Hormone concentrations and their ratios (means ± SD).

Variable	Group	Rest ^a,b^	Immediately Post-Exercise ^c,d,e^	15 Min Post-Exercise ^f^	30 Min Post-Exercise
TT (ng∙mL^−1^)	E1	4.97 ± 1.34	5.50 ± 1.58	4.40 ± 1.34	3.86 ± 1.14
	E2	4.57 ± 0.99	4.66 ± 0.94	4.01 ± 0.85	3.62 ± 0.73
FT (pg∙mL^−1^)	E1	12.75 ± 4.73	14.77 ± 6.41	12.67 ± 5.68	10.94 ± 5.23
	E2	12.75 ± 3.13	13.82 ± 3.67	12.62 ± 2.93	11.60 ± 2.45
C (ng∙mL^−1^)	E1	147.39 ± 32.15	189.76 ± 63.65	251.40 ± 80.55 *	203.71 ± 73.00 **
	E2	127.43 ± 38.56	185.77 ± 46.68	170.28 ± 73.85	135.46 ± 40.77
TT/C	E1	0.035 ± 0.011	0.032 ± 0.014	0.020 ± 0.014	0.023 ± 0.016
	E2	0.041 ± 0.020	0.027 ± 0.009	0.028 ± 0.014	0.029 ± 0.010
FT/C	E1	0.089 ± 0.033	0.083 ± 0.039	0.058 ± 0.034	0.063 ± 0.040
	E2	0.110 ± 0.043	0.077 ± 0.025	0.085 ± 0.038	0.090 ± 0.024

TT—serum total testosterone level; FT—serum free testosterone level; C—serum cortisol level; total testosterone/cortisol ratio (TT/C); free testosterone/cortisol ratio (FT/C). E1—playing six 30 s one-on-one (1 × 1) games with a 2 min rest period; E2—playing six 45 s 1 × 1 games with a 3 min rest period. ^a^ significantly greater TT than 30 min post-exercise. ^b^ significantly greater TT/C, FT/C than 15 min post-exercise, 30 min post-exercise. ^c^ significantly greater TT than 15 min post-exercise and 30 min post-exercise. ^d^ significantly greater FT than 30 min post-exercise. ^e^ significantly greater cortisol than at rest. ^f^ significantly greater cortisol than 30 min post-exercise. * significantly greater than immediately post-exercise and at rest. ** significantly greater than at rest.
